# Oxidized Proteins Differentially Affect Maturation and Activation of Human Monocyte-Derived Cells

**DOI:** 10.3390/cells11223659

**Published:** 2022-11-18

**Authors:** Ramona Clemen, Kevin Arlt, Lea Miebach, Thomas von Woedtke, Sander Bekeschus

**Affiliations:** 1 ZIK *plasmatis*, Leibniz Institute for Plasma Science and Technology (INP), Felix-Hausdorff-Str. 2, 17489 Greifswald, Germany; 2 Department of General, Thoracic, Vascular, and Visceral Surgery, Greifswald University Medical Center, Ferdinand-Sauerbruch-Str., 17475 Greifswald, Germany; 3 Institute for Hygiene and Environmental Medicine, Greifswald University Medical Center, Ferdinand-Sauerbruch-Str., 17475 Greifswald, Germany

**Keywords:** antigen uptake, calreticulin, inflammation, heat shock protein 27, heme oxygenase, high-mobility group box 1, interleukin-8, kINPen, reactive oxygen species, ROS, tumor microenvironment

## Abstract

In cancer, antigen-presenting cells (APC), including dendritic cells (DCs), take up and process proteins to mount adaptive antitumor immune responses. This often happens in the context of inflamed cancer, where reactive oxygen species (ROS) are ubiquitous to modify proteins. However, the inflammatory consequences of oxidized protein uptake in DCs are understudied. To this end, we investigated human monocyte-derived cell surface marker expression and cytokine release profiles when exposed to oxidized and native proteins. Seventeen proteins were analyzed, including viral proteins (e.g., CMV and HBV), inflammation-related proteins (e.g., HO1 and HMGB1), matrix proteins (e.g., Vim and Coll), and vastly in the laboratory used proteins (e.g., BSA and Ova). The multifaceted nature of inflammation-associated ROS was mimicked using gas plasma technology, generating reactive species cocktails for protein oxidation. Fourteen oxidized proteins led to elevated surface marker expression levels of CD25, CD40, CD80, CD86, and MHC-II as well as strongly modified release of IL6, IL8, IL10, IL12, IL23, MCP-1, and TNFα compared to their native counterparts. Especially IL8, heme oxygenase 2, and vimentin oxidation gave pronounced effects. Furthermore, protein kinase phospho-array studies in monocyte-derived cells pulsed with native vs. oxidized IL8 and insulin showed enhanced AKT and RSK2 phosphorylation. In summary, our data provide for the first time an overview of the functional consequences of oxidized protein uptake by human monocyte-derived cells and could therefore be a starting point for exploiting such principle in anticancer therapy in the future.

## 1. Introduction

Inflammation is a process of local defense against harm inflicted by tissue injury, infection, and irritants. Irrespective of acute or chronic inflammation, a central trait is the release of reactive oxygen species (ROS) into the tissue by professional phagocytes, such as neutrophils and macrophages [1]. There, ROS exhibit multiple purposes. At lower concentrations, they act as signaling molecules [2] to induce stress responses in the microenvironment rendering cells more resilient. For instance, evidence has shown that ROS are involved in immune cell activation and microenvironment modulation [3]. Moreover, ROS can also directly act as chemoattractants for additional phagocytes to migrate into the inflamed tissue [4]. At higher concentrations, ROS perform antimicrobial actions through their bacteriostatic and microbicidal effects. Another, although less focused, ROS effect is their contribution to oxidative posttranslational modifications (oxPTMs) of proteins. In the inflamed tissue microenvironment, cellular debris and proteins are constantly taken up by professional antigen-presenting cells (APCs), such as dendritic cells (DCs). APCs present cleaved protein samples (peptides) to T-cells in the draining lymph node to mount anti-infective and antitumor adaptive immune responses. In doing so, ROS are inevitably entangled in the immune cross-talk processes of antigen presentation.

Major ROS in the inflamed microenvironment are, for instance, superoxide, hydrogen peroxide, nitric oxide, and hypochlorous acid [5,6,7]. For some proteins, such as human serum albumin and other blood plasma proteins, it has been shown that ROS-mediated oxidation directly affects antigen presentation [8,9]. Such protein modifications are also known as advanced oxidation protein products (AOPPs) and serve as in vivo markers for chronic inflammation [10,11]. Not surprisingly, AOPPs have been linked to several types of diseases and conditions, such as chronic UV-B exposure and induction of cutaneous squamous cell carcinoma [12], urine-mediated nephropathy [13], and metabolic syndrome [14]. Hence, inflammation-produced ROS modify proteins and immune recognition, and links to autoimmunity are established, posing the question of how oxidatively modified proteins are perceived by key APCs, such as DCs. To this end, we selected a range of common laboratory as well as disease-relevant proteins and tested the effect of oxidation of those proteins on human monocyte-derived cells. Indeed, myeloid cells and partially stromal cells release a plethora of enzymatically generated ROS during inflammation, also in the tumor microenvironment [15]. For instance, nitric oxide synthases produce nitric oxide known to affect breast cancer cell states [16]. NADPH oxidases (NOX) are pivotal in the TME by generating superoxide, which can further dismutate to hydrogen peroxide (H_2_O_2_) [17]. Myeloperoxidase is known to generate hypochlorous acid [5], which further contributes to the generation of atomic and singlet oxygen as well as hydroxyl radicals [18]. It is known that these ROS are critical components in tumor progression, regression, and therapy resistance [19,20]. ROS have also been designated to be key in transitioning from chronic inflammation to carcinogenesis [21]. However, considering the many different types of ROS, it is currently impossible to recapitulate multi-ROS inflammation environments using traditional chemical or enzymatic techniques.

Gas plasma technology was employed to generate and test an array of oxidized proteins. Exposure to gas plasma, a partially ionized gas generated at body temperature, is known to transfer a multitude of ROS from the gas phase into a liquid. This concept can be exploited by treating protein suspensions with gas plasma, generating a variety of biomolecule modifications and oxPTMs [22,23,24]. By using gas plasma technology-derived multi-ROS mixtures, we mimicked the heterogenous ROS profile in inflamed tissues to a greater degree than single-ROS-type exposures would. Importantly, we used a well-characterized atmospheric pressure argon plasma jet with emission profiles and characteristics previously investigated to a thorough extent [25]. Among the species generated, the jet expels hydroxyl radicals, superoxide anions, singlet oxygen, and atomic oxygen [26,27,28].

In this work, more than a dozen proteins were exposed to gas plasma in vitro. Subsequently, each of the proteins was added to monocyte-derived cells. After prolonged culture, the surface marker and cytokine and chemokine secretion signatures were analyzed across a range of markers and molecules. For specific proteins, protein phosphorylation was assessed, and monocyte-derived cells were found to react to the oxidation of proteins in distinct ways in several instances, suggesting that such processes may play a part in physiology and pathology alike.

## 2. Materials and Methods

### 2.1. Monocyte Isolation and Culture

Peripheral blood mononuclear cells were isolated from buffy coats dedicated for research purposes and obtained from de-identified blood from the Institute of Transfusion Medicine (Greifswald University Medical Center, Greifswald, Germany) via Ficoll-Paque density gradient centrifugation as described before [29]. Erythrocytes were lysed (RBC lysis buffer; BioLegend, Amsterdam, The Netherlands), and CD14^+^ monocytes were separated via positive magnetic bead separation (BioLegend). Cell purity was assessed using flow cytometry, and viable cell counts were determined. 2 × 10^5^ cells were seeded in 24-well plates in 500 µL Roswell Park Memorial Institute medium (RPMI 1640; Corning, Kaiserslautern, Germany) containing 10% fetal bovine serum (Sigma-Aldrich, Taufkirchen, Germany), 1% L-glutamine (Corning), 1% penicillin and streptomycin (Corning), 800 international units (IU) human granulocyte-macrophage stimulating factor (GM-CSF; PeproTech, Hamburg, Germany) and 500 IU human interleukin (IL) 4 (IL-4; PeproTech) to generate immature human monocyte-derived cells. Cells were incubated at 37 °C with 95% humidity and 5% CO_2_.

### 2.2. Proteins and Oxidation

Monocyte-derived cells were incubated with the following native and oxidized (ox) proteins: cytomegalovirus gB (CMV; prospecTany, Rehovot, Israel), human papillomavirus 16 (HPV; prospecTany), Epstein–Barr virus (EBV; prospecTany), heat shock protein 27 (HSP27; Sigma-Aldrich), interleukin-8 (IL8) (BioLegend), insulin (Ins; Sigma-Aldrich), mumps virus nucleoprotein (Mumps; prospecTany), calreticulin (CRT; prospecTany), hepatitis B virus e-antigen (HBV; prospecTany), high-mobility group box 1 (HMGB1; Abcam, Cambridge, UK), heme oxygenase 1 (HO-1; prospecTany), heme oxygenase-2 (HO-2; prospecTany), vimentin (Vim; Sigma-Aldrich), collagen type II (Coll; Sigma-Aldrich), ovalbumin (Ova; Hyglos, Bernried, Germany), human albumin (Alb; Biotest Pharma, Dreieich, Germany), and bovine serum albumin (BSA; Sigma-Aldrich). Lyophilized proteins were reconstituted according to the manufacturer’s instructions and diluted in phosphate-buffered saline (PBS). For the screening, monocyte-derived cells were incubated with 10 ng/mL of the proteins, except for BSA, Alb, and Ova, which were added at a final concentration of 10 µg/mL. As a positive control, monocyte-derived cells were stimulated with 100 ng/mL lipopolysaccharide (LPS; Sigma-Aldrich), while the addition of PBS alone served as vehicle control. For treatment, 100 µL of PBS-containing proteins were pipetted in a 96-well U-bottom plate (Sarstedt, Sarstedt, Germany). Oxidation was achieved using the atmospheric pressure plasma jet kINPen (neoplas, Greifswald, Germany) [25] operated with argon (purity 99.9999%; Air Liquide, Bremen, Germany) as carrier gas with a flow rate of two standard liters per minute, a frequency of 1 MHz, and a dissipated power of 1 W. The protein suspension was exposed to the gas plasma-generating ROS for 30 s at a distance between the nozzle and the liquid surface of 10 mm in a non-conductive mode [30]. Gas flux-mediated evaporation of liquid was compensated by adding a predetermined amount of ddH_2_O. Cells and supernatants were collected 24 h later.

### 2.3. Flow Cytometry

Cells were collected in FACS tubes and washed three times with cold FACS washing buffer (Miltenyi Biotec, Bergisch-Gladbach, Germany). Surface marker expression was investigated by incubating the cells with fluorochrome-conjugated antibodies (Table 1) after incubation with Fc-block (BioLegend). Dead cells were excluded from analysis by adding iFluor860 (Biozol, Hamburg, Germany). Flow cytometry experiments were performed using a *CytoFLEX LX* device (Beckman-Coulter, Krefeld, Germany), and data were analyzed using *Kaluza* 2.1.3 software (Beckman-Coulter).

### 2.4. Chemokine and Cytokine Analysis

Supernatants of stimulated cells were collected after 24 h, and chemokines and cytokines were measured using bead-based cytokine detection technology according to the manufacturer’s instructions (BioLegend). The following targets were quantified against a 5-log standard curve: interleukin (IL) 1β, IL6, IL8, IL10, IL12p70, IL17A, IL18, IL23, IL33, interferon (IFN) α, IFNγ, monocyte-chemoattractant protein (MCP) 1, and tumor necrosis factor (TNF) α.

### 2.5. Protein Kinase Phospho-Array

At least 1 × 10^7^ monocyte-derived cells were cultured in the presence or absence of PBS, LPS, native or oxidized IL8, insulin, or Ova after 2 h starving. One hour later, cells were collected on ice and pelleted. Protein phosphorylation was analyzed using the Proteome Profiler Human Phospho-Kinase Array Kit (BioTechne, Wiesbaden, Germany) and Human/Mouse MAPK Phosphorylation Array (BioCat, Heidelberg, Germany) according to the manufacturer’s instructions. Signals were acquired after adding chemiluminescent reagent super signal WestPicoPlus (Thermo Fisher Scientific, Dreieich, Germany) in a chemiluminescence detection system (GE Healthcare, München, Germany).

### 2.6. Statistical Analysis

Graphing and statistical analysis were performed using *prism* 9.4.1 (GraphPad Software, San Diego, CA, USA). Comparison of surface marker expression and cytokine secretion was made using Mann–Whitney Test. Principal component analysis (PCA) was performed with normalized surface marker expression and cytokine concentration data sets. Levels of significance were indicated as follows: *p* < 0.05 (*), *p* < 0.01 (**), and *p* < 0.001 (***).

## 3. Results

### 3.1. Monocyte-Derived Cells Show Distinct Profiles upon Culture with 17 Different Proteins

**Figure 1:**
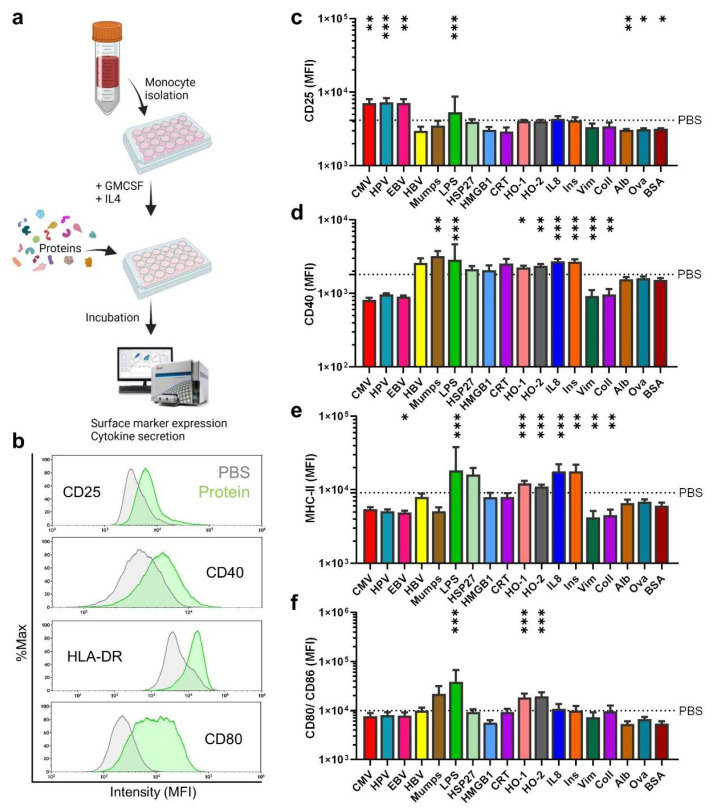
Monocyte-derived cells surface marker expression after uptake of different proteins. (**a**) scheme of the workflow to investigate the effect of proteins on monocyte-derived cells; (**b**) representative histogram of surface marker expression of CD25, CD40, HLA-DR, and CD80 investigated using flow cytometry 24 h after stimulation with the proteins; (**c**–**f**) quantification of activation marker CD25 (**c**), co-stimulatory receptor CD40 (**d**), maturation marker HLA-DR (**e**), and co-stimulatory and activation markers CD80/CD86 (**f**). Data are mean of six independent experiments, normalized to each corresponding native protein. Statistical analysis was performed using Mann–Whitney Test comparing each protein to PBS (* = *p* < 0.05, ** = *p* < 0.01, *** = *p* < 0.001).

**Figure 2:**
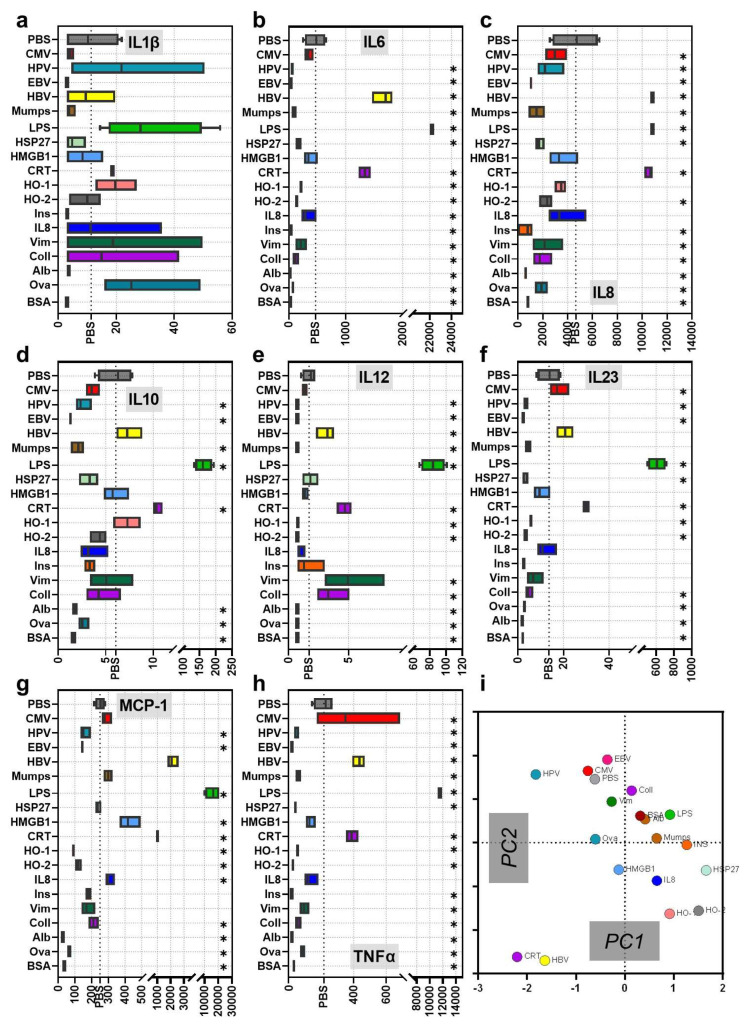
Secretion profiles of monocyte-derived cells incubated with different proteins. (**a**–**h**) supernatants of stimulated monocyte-derived cells were analyzed for pro-inflammatory and anti-inflammatory cytokines (in pg/mL) using a multiplex assay: IL1β (**a**), IL6 (**b**), IL8 (**c**), IL10 (**d**), IL12 (**e**), IL23 (**f**), MCP-1 (**g**), and TNFα (**h**); (**i**) principal component analysis of surface marker and secretion profiles. Data are mean of technical replicates of an assay with six independent experiments. Gas plasma-treated proteins were normalized to each corresponding native protein; LPS was normalized to PBS. Statistical analysis was performed using Mann–Whitney Test by comparing the proteins to PBS (*p* < 0.05, *p* < 0.01, *p* < 0.001 are grouped and given as *).

**Figure A1:**
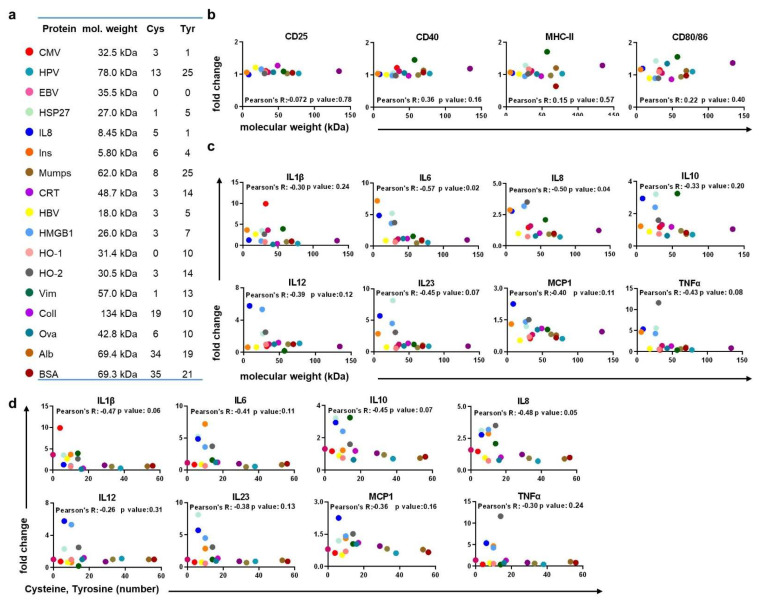
Correlation analysis between protein molecular weight and activation of myeloid-derived cells. (**a**) characteristics of proteins used in this study; (**b**) Pearson’s correlation between protein molecular weight (native; in kDa) and fold change expression of CD25, CD40, MHC-II, and CD80/86 on myeloid-derived cells after stimulation with oxidized proteins; (**c**,**d**) Pearson’s correlation between protein molecular weight (native; **c**) and cysteine and tyrosine numbers (**d**) and fold change cytokine release after stimulation of myeloid-derived cells with oxidized proteins.

At the site of inflammation, mononuclear phagocytes, such as immature dendritic cells and macrophages that derive from monocytes (moDC and moMac), engulf proteins and differentiate after activation to promote T-cell response. In this study, moDC and moMac are collectively referred to as monocyte-derived cells. Three signals from monocyte-derived cells are required to activate T-cells: antigen presentation via MHC, high surface expression levels of co-stimulating ligands, and cytokine secretion. CD14^+^ monocytes were isolated from blood donors and differentiated for three days using GM-CSF and IL4 before incubating them for 24 h with proteins (Figure 1a). Our protein library contained 17 proteins in the context of an infection (CMV, HPV, EBV, HBV, mumps, LPS), tumor and cellular stress response (HSP27, HMGB1, CRT, HO-1, HO-2, IL8), autoimmunity (insulin, collagen, vimentin, albumin) or others (ovalbumin, bovine serum albumin). Twenty-four hours after stimulation, supernatants were collected, and surface marker expression was analyzed by flow cytometry. The induction of activation and differentiation after stimulation with different proteins was determined by measuring CD25, CD40, HLA-DR, and CD80/ CD86, respectively (Figure 1b). Despite contextual similarities (e.g., disease-associated), incubation with the different proteins did not lead to a similar expression of the activation marker CD25 on monocyte-derived cells (Figure 1c). Unlike LPS, a microbe-associated molecular pattern (MAMP), none of the proteins resulted in a significantly enhanced expression of all four surface markers investigated (Figure 1d–f). However, several proteins led to marked changes, e.g., CMV, HPV, and EBV, resulting in a CD25 increase. IL8, Ins, Mumps, HO-1, and HO-2 showed no increased CD25 but CD40 and (except for Mumps) MHC-II levels, with the incubation with heme oxygenases even increasing co-stimulatory CD80/86 levels. CRT, HBV, HMGB1, Vim, Coll, Ova, Alb, and BSA mostly showed downregulation of all activation or differentiation markers on the cell surface. We next determined the concentration of IL6, TFNα, IL8, IL1β, MCP-1, IL10, IL23, and IL12 in the supernatant of stimulated monocyte-derived cells via multiplex assay (Figure 2a–h). LPS stimulation led to a significantly increased expression of all cytokines compared to PBS, in line with data on DC stimulation with bacteria [31,32]. Only CRT and HBV led to increased secretion of IL6, IL8, and TNFα. MCP-1 was secreted after stimulation with CMV, IL8, Mumps, CRT, HBV, and HMGB1, and is one of the key chemokines that regulate inflammation, migration, and infiltration of innate immune cells. For the polarizing cytokines IL1β, IL10, IL23, and IL-12, only LPS led to appreciably increased amounts. Nevertheless, several other proteins modulated their secretion to a significant extent. We performed principal component analysis, taking into account surface markers and cytokine secretion, and found that the proteins act on monocyte-derived cells independently of their origin or function (Figure 2i). Furthermore, the effects correlated only modestly with the molecular weight or amount of potentially oxidized cysteine and tyrosine of the native proteins (Appendix A: Figure A1a–d). Interestingly, some of the viral proteins tested (e.g., EBV, CMV, HPV) were located close to the PBS vehicle control. At the same time, the chaperone CRT and HBV—and to some extent also the heme oxygenases—had the most distinct effects compared to all other proteins investigated in our study.

### 3.2. Protein Oxidation Cultures Shape Monocyte-Derived Cells’ Maturation and Activation

**Figure 3:**
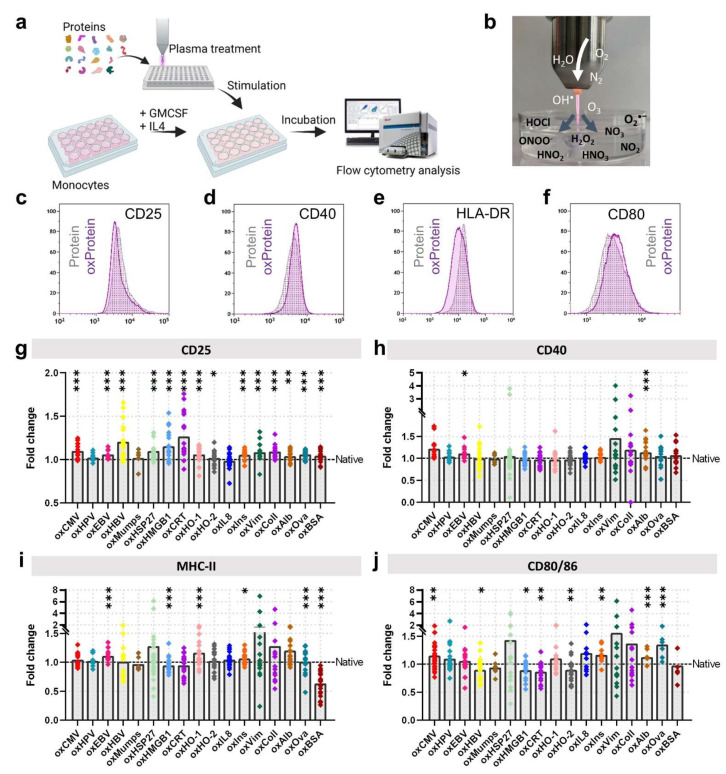
Gas plasma-oxidized proteins stimulate activation and maturation in phagocytes. (**a**) scheme of the experimental workflow; (**b**) gas plasma treatment of protein solutions; (**c**–**f**) representative histogram of surface marker expression of CD25 (**c**), CD40 (**d**), HLA-DR (**e**), and CD80 (**f**) investigated using flow cytometry 24 h after stimulation with proteins; (**g**–**j**) quantification of activation marker CD25 (**g**), co-stimulatory receptor CD40 (**h**), maturation marker HLA-DR (**i**), and activation markers CD80/CD86 (**j**). Data are mean of six independent experiments, normalized to each corresponding native protein. Statistical analysis was performed using Mann–Whitney Test comparing each protein to its native (* = *p* < 0.05, ** = *p* < 0.01, *** = *p* < 0.001).

**Figure 4:**
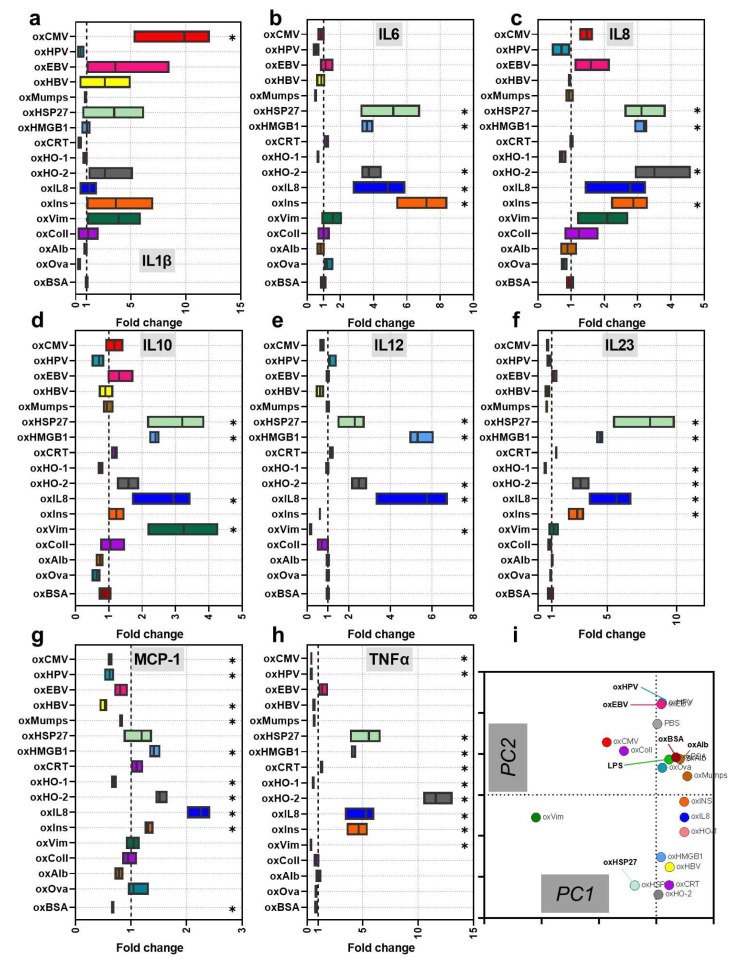
Secretion profiles of monocyte-derived cells incubated with oxProteins. (**a**–**h**) stimulated monocyte-derived cells’ supernatants were analyzed for cytokines using a multiplex assay: IL1β (**a**), IL-6 (**b**), IL8 (**c**), IL10 (**d**), IL12 (**e**), IL23 (**f**), MCP-1 (**g**), and TNFα (**h**); (**i**) principal component analysis of surface marker and secretion profiles. Data are mean of technical replicates of an assay with six independent experiments. Gas plasma-treated proteins were normalized to each corresponding native protein. Statistical analysis was performed using Mann–Whitney Test by comparing the oxidized proteins to the native (significances *p* < 0.05, *p* < 0.01, *p* < 0.001 are grouped and given as *).

Inflammation elicits potent ROS formation affecting not only cells but also proteins. To this end, the next question was how monocyte-derived cells respond to the oxidized version of each protein individually compared to the native (non-oxidized) protein samples (Figure 3a). Gas plasma technology was employed as a unique tool for generating multiple short-lived ROS/RNS simultaneously (Figure 3b). To learn about T-cell co-stimulation capacities, monocyte-derived cells were analyzed for several surface markers, such as CD25 (Figure 3c), CD40 (Figure 3d), HLA-DR (Figure 3e), and CD80 (Figure 3f) after incubation with 17 native or gas plasma-oxidized proteins. The maturation marker CD25 was consistently increased when cells were incubated with several of the oxidized protein compared to the native protein, albeit to a modest extent. In contrast, the co-stimulatory molecule CD40 showed only minor changes, e.g., with oxAlb (Figure 3h). MHC-II, in turn, was modestly but significantly increased with oxEBV, oxHMGB1, oxHO-1, oxIns, and oxAlb. At the same time, oxBSA led to significantly lower levels (Figure 3i). For CD80/CD86 (Figure 3j), oxidation of CMV, Ins, HBV, CRT, HMGB1, HBV, HO-2, Ova, and Alb led to significantly different expression levels. Subsequent secretion analysis revealed a major impact of protein oxidation on the release of cytokines and chemokines (Figure 4). Elevated IL6 levels were observed with oxidized HSP27, IL8, Ins, HO-2, and HMGB1 (Figure 4a). These, and CMV, HPV, CRT, HO-1, and Vim oxidation, significantly modified TNFα release (Figure 4b). For IL8, mostly an increase was observed with oxidized protein incubation, which was significant for HSP27, Ins, HMGB1, and HO-2 (Figure 4c). The same proteins also significantly elevated IL12 release (Figure 4d), while only oxCMV significantly changed IL1β levels (Figure 4e). IL23 was increased after stimulation with oxHsp27, oxIL8, oxIns, oxHMGB1, and oxHO-2 (Figure 4f). Upregulated IL10 is known to induce tolerogenic responses, and was observed for oxIL8, oxHMGB1, oxHSP27, and oxVim (Figure 4g). We further found increased MCP-1 concentration upon stimulation with oxHsp27, oxIL8, oxIns, oxCRT, oxHMGB1, and oxHO-2 (Figure 4h). In summary, for 17 oxidized proteins, responses were heterogenous for surface marker and secretion profiles. Oxidization of HSP27, CRT, and HO-2 seemed to generate the most pronounced effects in our study (Figure 4i).

### 3.3. Signaling Pathways in Monocyte-Derived Cells after Uptake of Oxidized Proteins

**Figure 5:**
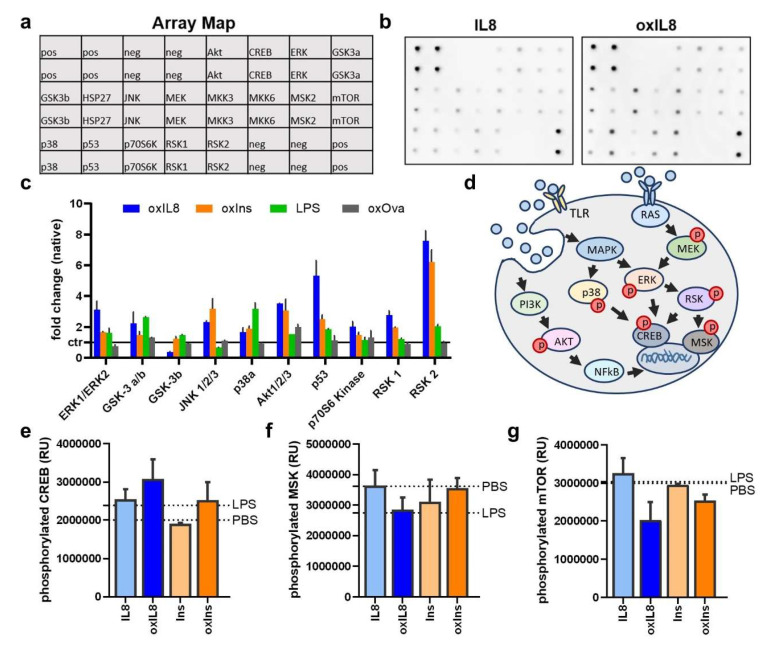
Gas plasma-treated insulin and IL8 reveal different modes of action in signaling cascades. (**a**,**b**) IL8 and insulin were chosen for phosphorylation assay (upper panel: antibody loading (**a**); lower panel: representative membranes (**b**)); (**c**) phosphorylation state of signaling proteins after stimulating monocyte-derived cells with oxIL8, oxIns, oxOva, or LPS; (**d**) simplified scheme of assignment of the proteins to different signaling pathways leading to phosphorylation of transcription factors CREB (**e**), MSK (**f**), and mTOR (**g**) using phospho-arrays. ctr = native protein/ PBS in case of normalized LPS. Data are mean of six to nine independent experiments.

Finally, the goal was to infer signaling pathway alterations induced by oxidized proteins in monocyte-derived cells by performing protein phospho-kinase screening (Figure 5a). Given the complexity of our screening and the large cell numbers needed for such an approach, we focused on two proteins, namely insulin and IL8, as these consistently induced acute phase secretion responses (Figure 4a–c). In addition, they were neither minimal nor maximal in the overall changes induced in monocyte-derived cells (Figure 4i). Ova was included as an exogenous, non-human antigen. After incubation for 1h, cells were lysed, and equal protein amounts were blotted (Figure 5b). OxIns and oxIL8 but not oxOva showed the most pronounced phosphorylation of p90 ribosomal S6 kinase (RSK) 1 and RSK2 compared to the native proteins (ctr). RSK1 and RSK2 were not or only marginally induced by LPS (Figure 5c). In contrast, LPS resulted in the phosphorylation of Glycogen synthase kinase 3 (GSK-3a/b) and p38a, which was not the case for oxIns and oxIL8. Furthermore, oxIns but not oxIL8 preferably induced stronger phosphorylation of Ras-extracellular signal-regulated kinase (Erk) and p53. Interestingly, except for pAkt, oxOva showed no overall change in the phosphorylation state of signal cascade proteins compared to the native Ova. Several downstream targets and transcription factors are described for the phosphorylation events investigated (Figure 5d). LPS, oxIL8, and oxIns increased phosphorylation of ERK1/2, leading to sequential activation of RSK and increased CREB phosphorylation (Figure 5e). The transcription factor MSK, which RSK induces as well, was downregulated in LPS and oxIL8-stimulated monocyte-derived cells but not with oxIns (Figure 5f). However, LPS did not lead to mTOR phosphorylation. In contrast, oxIL8 and oxIns showed decreased phosphorylation (Figure 5g). In summary, LPS, oxIL8, and oxIns preferably showed increased CREB activation, while mTOR and MSK were downregulated or unaffected.

## 4. Discussion

There is evidence that oxidized proteins correlate with immune cell activation and immune response, such as breaking immunological tolerance in autoimmune disorders [33,34]. Therefore, some oxidatively modified proteins and other proteins associated with oxidative stress are well-suited as biomarkers for various diseases [35,36,37]. During chronic inflammation in autoimmune disorders, acute inflammation in infection or injury, and oxidative stress, innate immune cells fight against structures by generating ROS and engulfing structures. This multi-ROS environment can be mimicked by gas plasma, an electron-impact and photon-driven technology, which enables the investigation of the immunological consequences of oxidatively modified proteins. In order to investigate the origin of the influences on an APC-mediated T-cell response, indications of different recognition and uptake mechanisms, activation signals, and maturation in monocyte-derived cells could be found by screening 17 oxidized proteins.

Extracellular ROS can induce oxidative posttranslational modifications (oxPTMs) in proteins at the amino acid level, leading to structural or conformational changes. Several factors, such as the position of the amino acid side chains and the peptide backbone as well as the site selectivity of the radical attack reflect the complexity of the ROS-induced protein oxidation [38]. The most commonly studied amino acids modified by reactive species are tyrosine and cysteine. Different oxidation states of cysteine are important for disulfide bonds that are often involved in protein structure and function. However, the thiol-disulfide exchange reactions are dynamic, and measuring the oxidation state of cysteine in proteins is still challenging [39]. Nevertheless, oxidative stress-mediated diseases are now well known, and some studies describe oxygen derivatives in proteins from patients. For instance, cystine (cys-cys) was identified as a biomarker for oxidative stress and the effectiveness of antioxidant treatment [40]. Another study has determined cysteine/cystine redox potential and provided evidence of a correlation between CD4^+^ T-cell counts and paralleled oxidation [41]. In plasma medicine, the derivates cystine, sulfinic acid (Cys-SO_2_H), and sulfonic acid (Cys-SO_3_H) were observed after gas plasma treatment of cysteine [42], and oxidative derivates on tyrosin were identified in proteins of gas plasma-treated wounds [43]. In our study, oxPTMs were not investigated since we did not measure any correlation between Cys + Tyr and immunological effects (Figure A1), and it remains doubtful that immune cells recognize an oxidized amino acid specifically during an inflammatory reaction. Added to this is the subsequent difficulty of assigning effects in a complex system (e.g., animal experiments, patient studies) to the allegedly specifically induced changes.

Our study gives a rough overview of changes in the surface marker expression and cytokine secretion in monocyte-derived cells after recognizing oxidized proteins. Interestingly, activation of monocyte-derived cells was not necessarily associated with increased cytokine secretion. For instance, oxIL8 and oxIns did not show notable changes in CD25, CD40, and MHC II expression, except for a slight upregulation of CD80/86 (Figure 1), while both oxidized proteins resulted in significantly increased pro-inflammatory cytokine secretion (Figure 2).

When interpreting the data, it must also be taken into account that experiments with human monocyte-derived cells from donors show a high variance due to, e.g., infection-related background activity, unique immune system, or previous illness of the individual donor. Such polymorphisms have previously been demonstrated in DC responses to TLR-mediated stimuli, which have also been suggested to be due to differential susceptibility to infectious pathogens or autoimmune diseases within the human population [44]. However, we do not see large deviations in our data compared to such “high” and “low” responders. In addition, it should be mentioned that some proteins lead to increased or decreased marker expression and/or cytokine secretion even in their native state.

Minor differences between oxidized vs. native proteins can still influence the immune response, e.g., interactions with other immune cells could lead to recruitment to differences in leukocyte recruitment. For instance, we saw only minor changes for CD40 after incubation with oxidized EBV and Vim. Inflammation also drives the upregulation of CD40L on CD4^+^ T-cells, thereby turning CD40-expressing DCs into more effective APCs. In combination with increased secretion of IL10 and downregulated IL12, oxidized vimentin seems to induce a tolerogenic immune response—which was unexpected, as modified vimentin is known as autoantigen and autoantibody production [45,46]. Interestingly, oxidized collagen type II showed increased surface marker expression (CD25, CD40, MHC-II, and CD80/CD86) similar to oxVim, but IL10 secretion was not increased. Those results suggest that oxColl is recognized as a more potent autoantigen leading to autoimmunity and autoantibody production, as described previously [33,47].

Other investigated proteins are not associated with autoimmune disorders, such as calreticulin (CRT) and high-mobility group box 1 (HMGB1). However, they have been identified as DAMPs and secreted as danger signals from dead or stressed cells to elicit inflammation and subsequent immune responses. Therefore, it is not surprising that in our study DCs secrete pro-inflammatory cytokines when stimulated with oxHMGB1, as they can signal ROS-induced redox stress. Previously, HMGB1 was shown to promote DC maturation (TNF, IFNα) when cells were activated with Toll-like receptors (TLR)-9 stimulating CpG [48]. In line with those results, in our study, we see increased TNFα secretion, suggesting oxHMGB1 stimulates TLR-9 as well. However, oxidized HMGB1 (oxidation of C at position 106) was previously shown to have less immunogenic properties in vivo [49], which would explain increased IL10 secretion and no activation (less CD80/86). These conflicting results underscore the need for further studies of immune cell response and in vivo experiments.

Strikingly, a generalized activation could not be observed in our study, raising the question of identifying factors determining the immunogenicity of oxidatively modified proteins. However, delineating structural and biochemical requirements or treatment-related parameters is complex as production kinetics, and specificity of ROS are hard to control, while oxidative modifications are further challenging to track and require versatile, costly detection methods. This study’s increased activation of phagocytes points out an inherent damage-associated molecular patterns (DAMPs) activity of oxidized proteins. Similar observations have been reported for advanced glycosylation end-products (AGE) on naturally occurring carbohydrates activating APCs via scavengers, mannose receptors, RAGE, and galectin-3 [50]. Likewise, protein aggregation affects immunogenicity [51] and has been shown to increase the frequency and amount of antigen uptake by DCs previously [52]. Differences between the proteins investigated might likely be linked to their physiological function and different structures, amino acid composition, and sequences. Here, the correlation of cytokine and chemokine release profiles and molecular weight of the respective native proteins revealed a negative relation in tendency for all proteins, which was significant for the release of IL6 and IL8 in the present study. It is conceivable that this is attributed to a diminished uptake of antigens at a higher molecular weight [53] that cannot be considerably enhanced by protein oxidation. Second, a reduced surface-to-volume ratio of larger proteins might hamper effective oxidation.

Various receptors enable DCs to sense diverse extracellular stimuli, followed by activation, maturation, and cytokine secretion, driving differentiation and expansion of T helper cell subtypes [54]. Once a ligand binds to its receptor, signaling pathways are induced, resulting in the activation of transcription factors. The signaling p38, Erk, and JNK have been reported to be relevant for DC maturation by regulating surface marker expression, cytokine expression, and allostimulatory capacity (reviewed in [55]). The most focused receptors are TLRs that have broad specificity for conserved molecular patterns and enable DCs to discriminate between stimulatory molecules. TLR comprises different subclasses that recognize various conserved structures, such as TLR4 binding to LPS, TLR5 binding to bacterial flagellin, TLR9 for CpG-containing DNA, and TLR2 binding to bacterial lipopeptide. In line with previous studies [56], LPS stimulation induced phosphorylation of p38 and ERK. However, stimulatory molecules for distinct TLR showed different effects on p38 and ERK [57,58,59], which was also observed in our study after stimulating monocyte-derived cells with oxIL8, oxIns, or oxOva, compared to LPS. Therefore, we suggest that LPS and oxProteins act as other ligands, and there is no conserved structure for recognizing oxidized targets. This hypothesis was confirmed by observing different effects on transcription factors and, of course, by the previous differences in surface marker expression and cytokine secretion.

For instance, HOCl-modified Ova stimulates and activates APCs [60,61]. Other studies have shown that HOCl-treated ovalbumin (Ova) leads to increased APC-dependent T-cell activation of lymphocytes from transgenic animals [62]. However, HOCl is a long-lived species formed by the inversion of H_2_O_2_ during an inflammatory response and is not nearly as reactive as H_2_O_2_. In contrast, short-lived species interact faster with a target structure, such as a protein. Physical gas plasma technology generates various short-lived and long-lived ROS, and its physical, medical, and biological applications are versatile [63,64,65]. This technology is suitable for inducing protein modifications through a ROS cocktail [23], leading to altered functionality and showing indications for altered immunogenicity [66,67,68,69,70]. For the first time, we have recently shown solid in vitro and in vivo evidence for altered immunogenicity of gas plasma-treated proteins linked to oxidative posttranslational modifications (oxPTM) [71,72]. However, it is still unclear if oxPTMs are specifically recognized by APCs, leading to altered signaling pathways.

## 5. Conclusions

Reactive oxygen and nitrogen species are important in pathophysiological conditions because they are generated by immune cells during an inflammatory response, thereby inducing oxidative modifications to proteins. Innate immune cells take up such extracellular oxidized proteins, become activated, and maturate to support an immune response. Using gas plasma technology as a tool to generate various ROS simultaneously, this study identified altered phenotypic alterations and cytokine secretion profiles in monocyte-derived cells after stimulation with 17 different oxidized proteins and LPS. IL8 and insulin were chosen to investigate signaling pathways in monocyte-derived cells. The observed changes in signaling pathways, activation/ maturational surface markers, and cytokine response help refine the complexity of oxidized proteins in an inflammatory microenvironment. Taken together, the results show different immunogenicities of exogenous ROS-induced oxidized proteins—whereby each protein, each species, each modification, but no differences in protein concentrations leads to an individual reaction—and functional consequences in activating myeloid cells can influence the course of the immune response.

## Figures and Tables

**Table 1:** Antibodies used in this study.

Ligand	Fluorescent Marker	Clone	Supplier
HLA-A,B,C	AF700	W6/32	BioLegend
CD40	APC	HB14	BioLegend
HLA-DR	APC-cy7	L246	BioLegend
CD69	AF488	FN50	BioLegend
CD45RA	PerCP/Cyanine5.5	HI100	BD Biosciences
CD80	PE	2D10	BioLegend
CD86	PE	BU63	BioLegend
CD11C	PE-Cy7	S-HCL-3	BioLegend
CD25	AF488	BC96	BioLegend
CD83	PerCP/Cyanine5.5	HB15	BioLegend
CD14	BV650	M5E2	BioLegend

## Data Availability

The underlying data of this study are available from the corresponding author upon reasonable request.
